# Incidental Cytodiagnosis of Carotid Body Tumour on Fine Needle Aspiration Cytology

**DOI:** 10.7759/cureus.54817

**Published:** 2024-02-24

**Authors:** Sreetama Mukherjee, Arvind Bhake

**Affiliations:** 1 Pathology, Jawaharlal Nehru Medical College, Datta Meghe Institute of Higher Education and Research, Wardha, IND

**Keywords:** histopathology, head and neck mass, paraganglioma, fine needle aspiration cytology, carotid body tumour

## Abstract

A carotid body tumor is a rare form of neoplasm that arises near the carotid artery bifurcation and it has an incidence rate of less than 0.3 per 100,000 population. The low incidence rate of such tumors is due to their origination from the paraganglion cells which is relatively uncommon as compared to other forms of tumor. Here we present an incidental and unusual cytodiagnosis of carotid body tumors. A 45-year-old male presented to the surgery outpatient department with swelling in the left-sided anterior region of the neck. The swelling was gradually progressing over three years and was insidious. Clinically the swelling was 3 cm x 3 cm in size and the patient complained of pain in the last two months which was intermittent. The patient was sent to the cytopathology section for fine needle aspiration cytology with the clinical diagnosis of tubercular lymphadenopathy. The patient underwent fine needle aspiration cytology by 26 SW needle by standard institutional protocols. The cytodiagnosis of “Paraganglioma/ Carotid Body Tumour” was offered. Histomorphological features at excision were consistent with carotid body tumors (paraganglioma). The sections of the tumor immunohistochemically were positive for neuron-specific enolase. The incidental cytodiagnosis of carotid body tumors is reported in the literature sparsely. This case is presented for the cytomorphology of carotid body tumor which is unfamiliar to the reporting pathologist because of its rare occurrence.

## Introduction

Carotid body tumors are slowly growing neoplasms rising from carotid bodies located at the posteromedial aspect of the carotid artery bifurcation. The largest group of paraganglia in the head and neck is called the carotid body, and it is situated on the medial side of the carotid bifurcation bilaterally. Carotid body tumors sometimes referred to as paragangliomas or chemodectomas, are rare neuroendocrine tumors that arise in glomus cells derived from the neural crest of the embryo near the carotid bifurcation. They are rare neck tumors confused with a cervical neck mass and form a wide clinical differential diagnosis. The incidence is around 0.3 per 100,000 population and 0.5% of total head and neck masses [1]. The cells of carotid body tumors are neuroendocrinal by function and possess chemoreceptors. Fine needle aspiration cytology diagnosis of carotid body tumors is infrequently reported in the literature [1, 2, 3].

The present case report describes the incidental finding of the carotid body tumor with the help of fine needle aspiration cytology which was not confirmed in the clinical diagnosis [4, 5, 6]. Fine needle aspiration cytology is contra-indicated for carotid body tumors as it can lead to hypertensive crisis [7, 8]. This case report contains the comprehensive details of cytomorphological features of carotid body tumors along with their histopathology and the findings of radio imaging technique in a 45-year-old male.

## Case presentation

**Figure 1:**
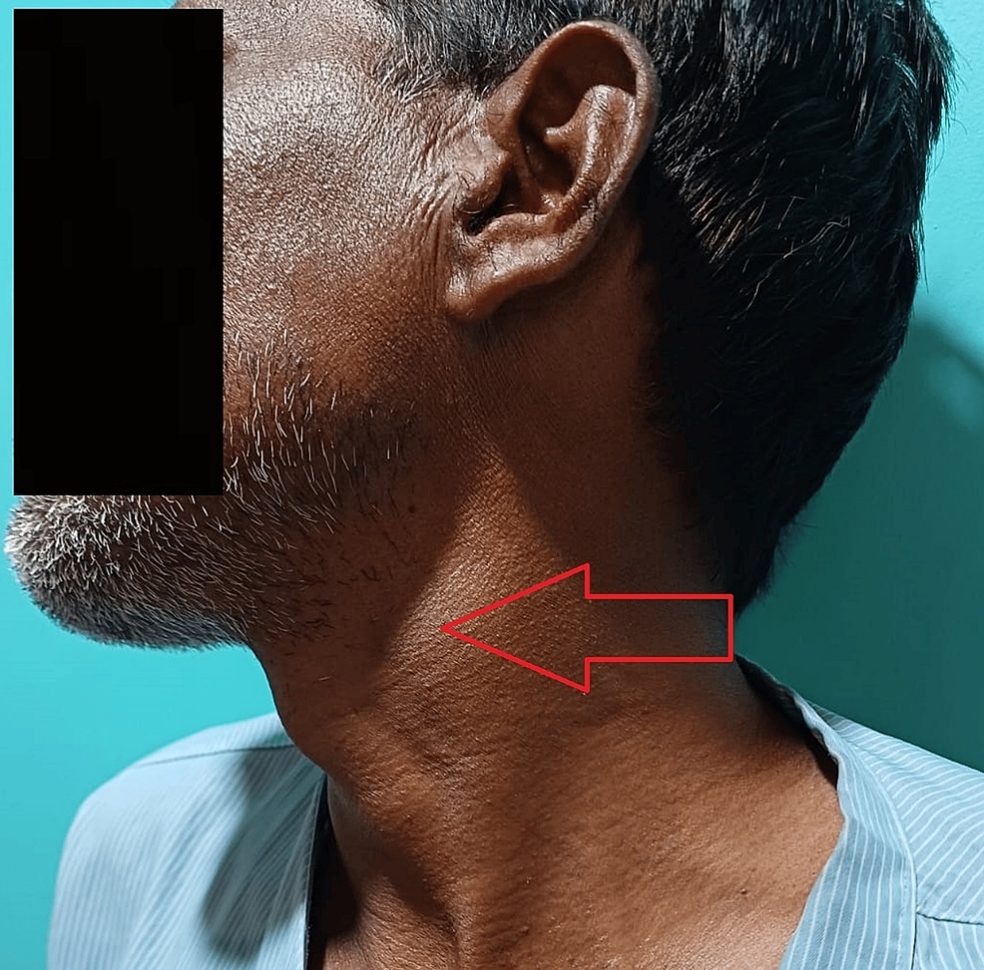
Clinical photograph of tumor swelling in the anterior triangle.

A 45-year-old male presented to the surgery outpatient department with swelling in the left-sided anterior region of the neck (Figure 1). The swelling was gradually progressing over three years and was insidious. Clinically the swelling was 3 cm x 3 cm in size and the patient complained of pain in the last two months which was intermittent. The pain was not associated with any aggravating factors and no relieving factors for it. The skin over the swelling was normal. Sinuses and discharge were not present. The swelling was non-tender and had limited mobility in all directions. The fluctuation test was negative and did not adhere to the underlying structure.

The patient was sent to the cytopathology section for fine needle aspiration cytology with the clinical diagnosis of tubercular lymphadenopathy. The clinical examination before fine needle aspiration carried out by a performing pathologist revealed feeble transmitted pulsation. The patient was normotensive. His personal history was unyielding to his present complaint of swelling.

The patient underwent fine needle aspiration cytology by standard institutional protocols by 26 SWG (Standard Wire Gauge) needle. The material yielded on fine needle aspiration cytology was blood mixed. The smears were stained with Papanicolaou and May-Grunwald-Giemsa (MGG) stain. 

**Figure 2:**
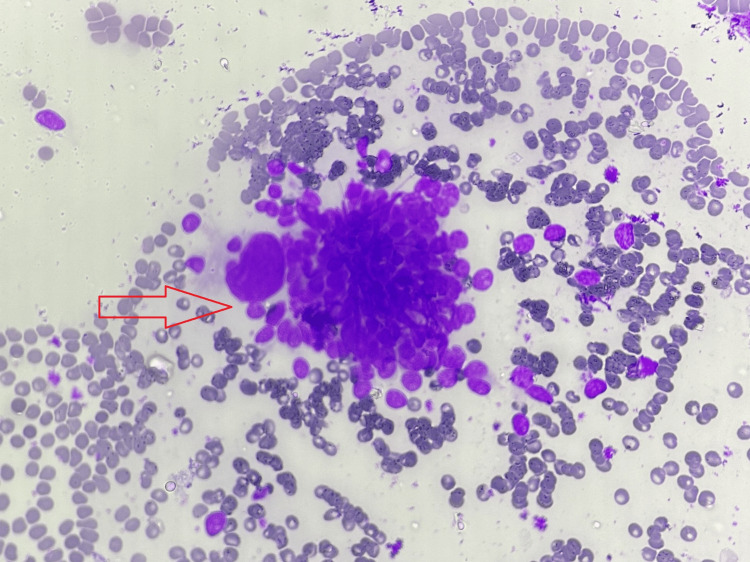
Fine needle aspiration cytology (FNAC) of carotid body tumor. The smear shows cell ball-like arrangement (Zellballen) of small to intermediate size cell (MGG stain) 10X MGG: May-Grunwald-Giemsa

**Figure 3:**
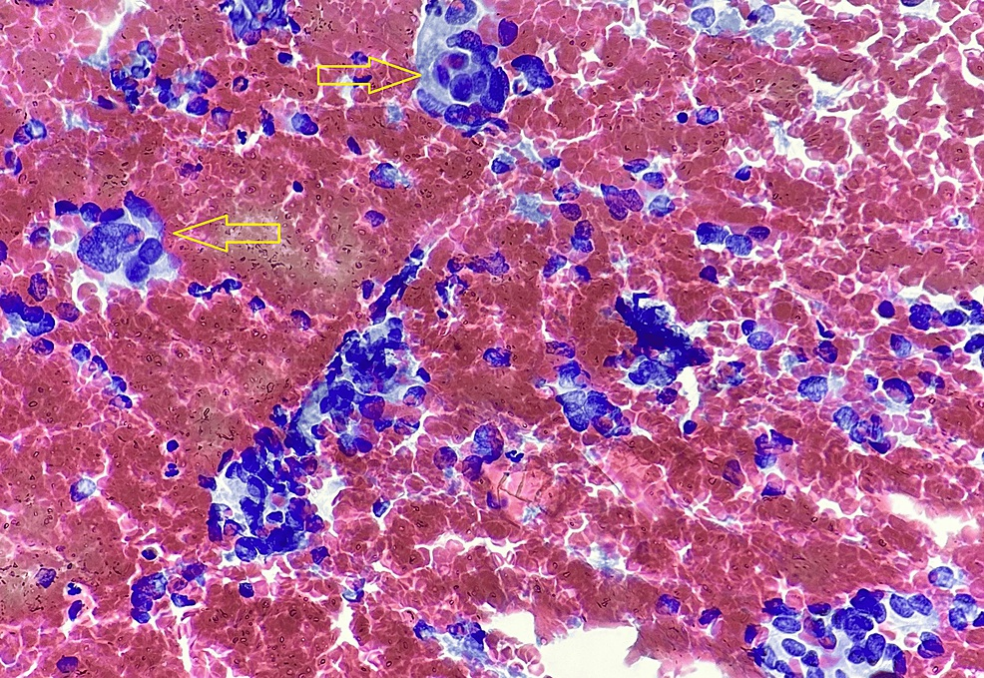
Papanicolaou stain, 40X - the smear shows multiple, small nested balls with nuclei showing chromatin (salt and pepper chromatin) of neuroendocrinal features

Smears with mild to moderate cellularity showed intermediate-size cells, predominantly polygonal cells placed in organized ball-like arrangements in small sheets with attempted pseudo-acinus and isolated ones (Figure 2). These cells were intervened in between by spindle cells. A few of the cell sheets showed central nuclear crowding. The cells carried hyperchromatic nuclei with moderate nuclear enlargement, pleomorphism, and microvesicular chromatin pattern. A few of the cells showed large, stand-out nuclei. A few showed binucleation, and a few nuclei showed micronuclei. The cytoplasm of many cells showed peripheral magenta-coloured granules (Figure 3). The background showed many bare nuclei with micronuclei and more open chromatin, nuclear streaks, and nuclear smudging admixed in hemorrhagic material. The cytodiagnosis of “Paraganglioma/Carotid Body Tumor” was offered. No cytologically malignant cells were seen.

**Figure 4:**
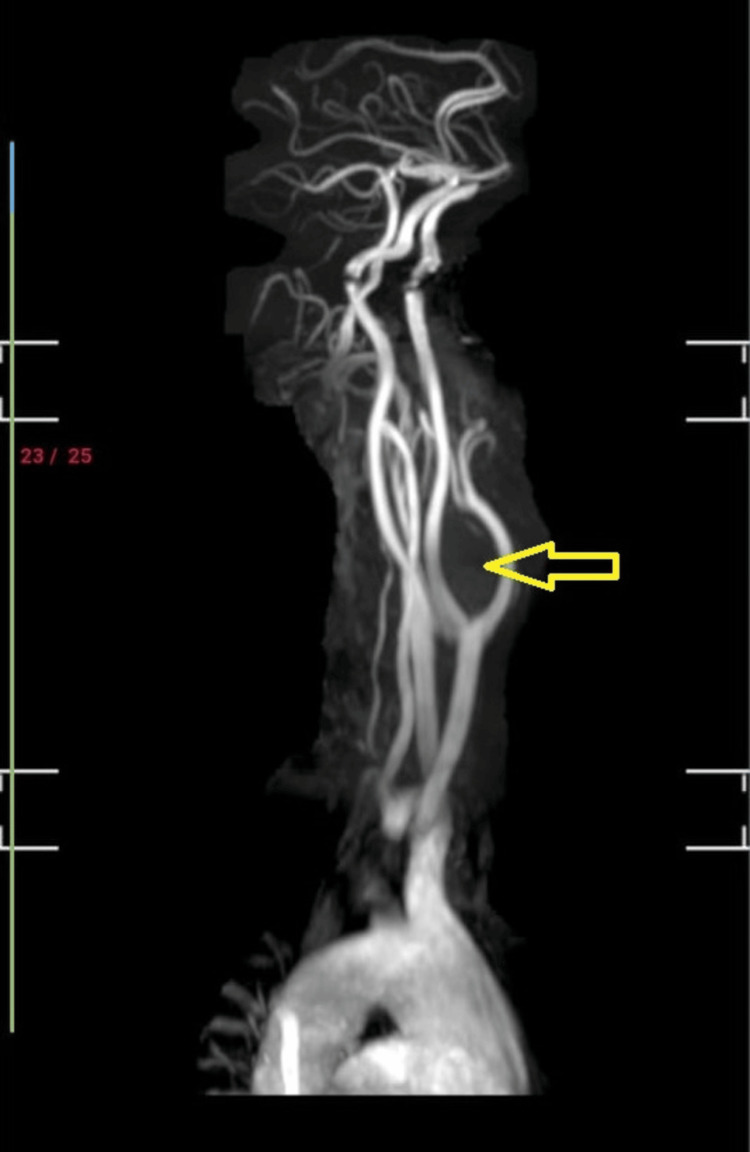
Magnetic Resonance Angiography revealed a soft tissue mass in the carotid space causing a mass effect in the form of splaying of the internal and external carotid artery.

**Figure 5:**
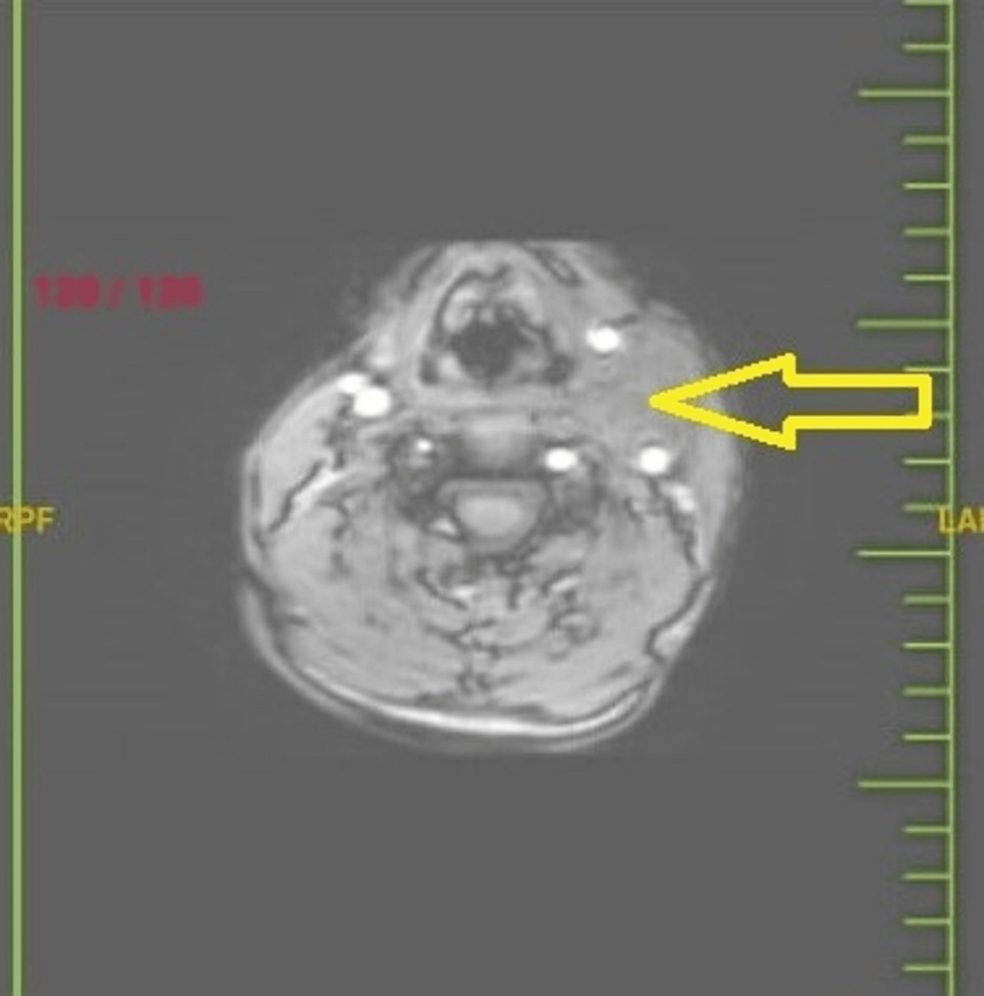
MRI brain showed a soft tissue mass lesion in the carotid space causing a mass effect suggestive of a carotid body tumor.

Post fine needle aspiration cytology diagnosis, the patient was referred for Magnetic Resonance Imaging (MRI) and Colour Doppler studies. Magnetic Resonance Angiography (MRA) revealed a soft tissue mass lesion noted in the carotid space, causing a mass effect in the form of splaying of the carotid vessels, that is, internal and external carotid artery (Figure 4). At carotid bifurcation, the internal carotid artery was displaced laterally, and the external carotid artery was displaced medially. Hypoplastic V2, V3, and V4 segments on the right side and the V4 segment on the right side were not seen joining the vertebral artery. The bilateral internal carotid left vertebral circle of Willis and its branches appeared to be normal in course and caliber. The impression on the MRI brain was of a soft tissue mass lesion in the carotid space causing a mass effect, suggesting a carotid body tumor (Figure 5). The Colour Doppler study showed a 3.2 cm x 3.6 cm mass abetting the internal and external carotid artery just cranially at the bifurcation of the left carotid artery. The mass showed increased internal vascularity.

**Figure 6:**
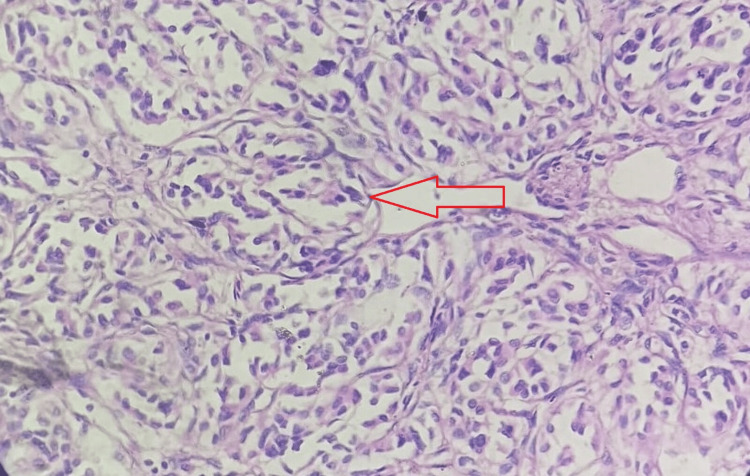
Hematoxylin & Eosin stain, 40X - the section shows a well-developed ‘Zellballen’ growth pattern, basophilic granular cytoplasm and round hyperchromatic nuclei with dispersed chromatin.

**Figure 7:**
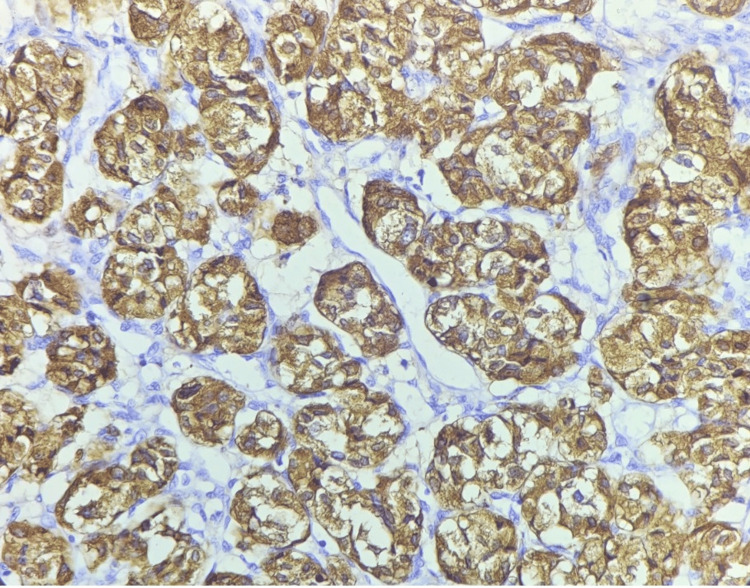
Photomicrograph of carotid body tumor showed strong positive reactivity with chromogranin staining by immunohistochemistry.

**Figure 8:**
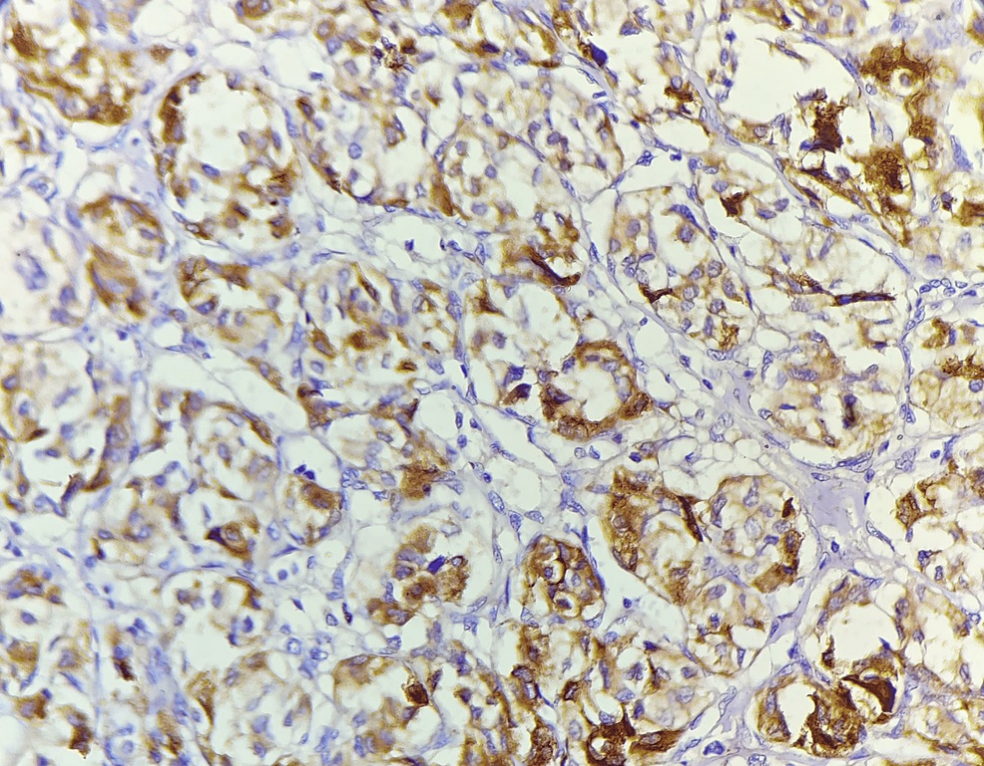
Photomicrograph of carotid body tumor showed positive reactivity with synaptophysin staining by immunohistochemistry.

The present case underwent excision of the tumor. The excised tumor mass appeared to be a single, globular, fleshy brown, measuring 2.5 x 2 x 1.5 cm and was well circumscribed and encapsulated. The excised tumor mass cut section showed wide areas of hemorrhages and was fleshy. The section showed cells were arranged in a well-developed ‘Zellballen’ growth pattern, basophilic granular cytoplasm, and round hyperchromatic nuclei with dispersed chromatin (Figure 6). The histomorphological examination of the tumor mass was consistent with the diagnosis of a carotid body tumor (Paraganglioma). The sections of the tumor paraffin block immunohistochemically were positive for chromogranin, synaptophysin, and neuron-specific enolase (Figures 7, 8). 

## Discussion

The differential diagnosis of lateral neck masses can reveal a wide range of possibilities which includes tubercular lymphadenitis branchial cleft cyst or metastatic carcinoma, lymphoma, schwannoma, or carotid body tumor. Until the clinical examination is highly suggestive of the swelling being the carotid body tumor, it remains unsuspected [4, 5, 9].

The carotid body tumor rises from the specialized neural crest cells which are placed at carotid body bifurcation. The masses of carotid body tumors are usually painless, slowly enlarging with or without signs and symptoms of excessive catecholamine secretion [10-12].

Most of the cases reported in the literature for the diagnosis of carotid body tumors by fine needle aspiration cytology were incidental and their clinical diagnosis was otherwise. These cases were sent for fine needle aspiration cytology much before the radio imaging investigation such as ultrasonography, color doppler, CT, or MRI. The present case was one like the above situation where the patient was referred for fine needle aspiration cytology before being investigated radiologically [1, 4, 7, 8, 9, 10, 11, 12].

It’s controversial to perform the procedure of fine needle aspiration cytology in a patient where a carotid body tumor is either clinically suspected or suggested on radiological investigation. The risk of hemorrhage during the procedure of fine needle aspiration cytology may lead to a sudden increase in blood pressure which may be life-threatening. Many authors have highlighted this complication of the fine needle aspiration cytology reviewed for the present work. The sudden increase in blood pressure in the procedure of fine needle aspiration cytology of carotid body tumors is attributed to the release of norepinephrine from the tumor. The present case for its later cervical mass has a diagnosis of tubercular lymphadenopathy. However, the procedure of fine needle aspiration cytology did not give rise to any such condition of syncope [7, 8].

The well-documented cytomorphological features of carotid body tumors have been reported by numerous authors (Nagiredla P et al [8], Dukkipati K et al [9], Tessy PJ et al [11]). The smears usually displayed blood and tumour cells which were either in sheets or arranged in groups of clusters or isolated ones. These cells in the sheets seemed to be intervened by spindle endothelial cells and there was nuclear crowding in the few sheets of cells These cells were large, polygonal, or ovoid with moderate to abundant cytoplasm and mild to moderate anisonucleosis. The cytomorphology of carotid body tumors described in various articles is similar to the cytologic features of our case [7, 8, 9, 10, 11, 12]

The studies reviewed from the present time also stated that the degree of nuclear pleomorphism, prominent nucleoli, clumped chromatin or significant anisonucleosis seen on cytologic preparation does not imply malignancy in the lesion of the carotid body tumor [11]

After cytodiagnosis, the present case was referred for Doppler, ultrasonography, and MRI, and all these investigations suggested the lesion to be a carotid body tumor. The histopathological section of the tumor paraffin block of the present case also investigated immunohistochemistry for neuron-specific enolase, the result of which was positive. This supported the diagnosis of carotid body tumor. A few of the authors (Carillo AM et al [5], Masilamani S et al [10]) have carried out immunohistochemistry techniques for chromogranin, S100 and neuron-specific enolase in confirmation of the diagnosis of carotid body tumors [5, 10]

## Conclusions

The experience with the present case confirms that the fine needle aspiration cytology of the carotid body tumor is incidental. Therefore, such tumors which are clinically suspected of carotid body tumors should undergo color Doppler, CT/MRI studies to confirm their nature that may avoid the needling of the tumor and its consequences.

However, the incidentally aspirated carotid body tumors show cytomorphology consistent with carotid body tumors. Such a diagnosis provides ease of a preoperative diagnosis for a surgeon for surgical excision of such tumors.

## References

[1] Handa U, Kundu R, Mohan H (2014). Cytomorphologic spectrum in aspirates of extra-adrenal paraganglioma. J Cytol.

[2] Wieneke JA, Smith A (2009). Paraganglioma: carotid body tumor. Head Neck Pathol.

[3] Sallom M, Al Laham O, Ghannam E, Ghannam M, Mohammad A (2023). Unilateral synchronous masses of the neck revealing a malignant Carotid Body Tumor: A case report and literature review. Int J Surg Case Rep.

[4] Vazquez Salas S, Pedro K, Balram A (2022). Head and neck cystic lesions: a cytology review of common and uncommon entities. Acta Cytol.

[5] Carillo AM, Franca RA, Modica R (2023). Interventional cytopathologist can successfully combine ultrasonographical and microscopic skills to narrow the differential diagnosis in fine needle aspiration of neck paraganglioma. Cytopathology.

[6] Conrad R, Yang SE, Chang S, Bhasin M, Sullivan PS, Moatamed NA, Lu DY (2018). Comparison of cytopathologist-performed ultrasound-guided fine-needle aspiration with cytopathologist-performed palpation-guided fine-needle aspiration: a single institutional experience. Arch Pathol Lab Med.

[7] Majumdar A, Jana A, Jana A, Biswas S (2014). Carotid body paraganglioma fine-needle aspiration cytology. J Med Trop.

[8] Nagiredla P, Tummidi S, Patro MK (2019). Carotid body tumor diagnosed by on-site FNA: a case report. Indian J Surg Oncol.

[9] Dukkipati K, Kumar OS (20151). Fine-needle aspiration cytology diagnosis of paraganglioma (carotid body tumor). J NTR UnivHealth Sci.

[10] Masilamani S, Duvuru P, Sundaram S (2012). Fine needle aspiration cytology diagnosis of a case of carotid body tumour. Singapore Med J.

[11] Tessy PJ, Jojo A, Sreejesh S, Krishnan SA (2015). Role of FNAC in the diagnosis of carotid body paraganglioma. Peoples J Sci Res.

[12] Mondal P, Basu N, Gupta SS, Bhattacharya N, Mallick MG (2009). Fine needle aspiration cytology of parapharyngeal tumors. J Cytol.

